# Association between Consumption of Dietary Supplements and Chronic Kidney Disease Prevalence: Results of the Korean Nationwide Population-Based Survey

**DOI:** 10.3390/nu15040822

**Published:** 2023-02-05

**Authors:** Yina Fang, Hwasun Lee, Serhim Son, Sewon Oh, Sang-Kyung Jo, Wonyong Cho, Myung-Gyu Kim

**Affiliations:** 1 Department of Internal Medicine, Korea University Anam Hospital, Seoul 02841, Republic of Korea; 2 Department of Biostatistics, Korea University College of Medicine, Seoul 02842, Republic of Korea

**Keywords:** chronic kidney disease, dietary supplements

## Abstract

Despite the enormous global market of dietary supplements, the impact of dietary supplements on kidney disease is still unclear. Based on the National Health and Nutrition Examination Survey from 2015 to 2017, this study evaluated the association between dietary supplement and chronic kidney disease (CKD) in 13,271 Korean adults. Among the dietary supplements, vitamin and mineral intake was the highest at 61.41%, followed by omega-3 fatty acids at 11.85%, and ginseng at 7.99%. The prevalence of CKD was significantly higher in those who consumed amino acids and proteins, ginseng and red ginseng, and herbal medicine (plant extract)-berries than in those who did not. Conversely, patients who consumed probiotic supplements had a significantly lower prevalence of CKD than those who did not. In the population without CKD risk factors or history of CKD, the prevalence of CKD was high in the group consuming ginseng and red ginseng. After adjusting for covariates, the herbal medicine (plant extract)-berry group showed an independent association with CKD incidence. In conclusion, it is suggested that dietary supplements may affect kidney function. Further large-scale cohort studies are required to elucidate the exact effects of each dietary supplement on CKD.

## 1. Introduction

Over the past decades, chronic kidney disease (CKD) has affected 10–15% of the population worldwide, and paralleling epidemics of hypertension and diabetes, the number of CKD patients has increased rapidly in recent years, making a significant impact on the global health burden [1,2,3,4]. Owing to the high prevalence, morbidity rates, and medical costs of CKD, prevention and optimal management of the disease is an important public health issue.

However, since there is no effective kidney-targeting drug that can inhibit the progression of CKD other than treatment of underlying diseases such as hypertension, hyperglycemia, and dyslipidemia and monitoring of complications, interest in the effect of nutrition or dietary supplements on kidney disease is increasing. Diets such as protein restriction diet, the Mediterranean diet, and plant-based diets are currently being investigated for their potential roles in delaying CKD progression.

Today, there are thousands of dietary supplements available on the market, including vitamins and minerals, plant ingredients and extracts, proteins and amino acids, omega-3 fatty acids, probiotics, and prebiotics [5,6,7]. Some research has indicated that dietary supplements can compensate for the nutritional deficit derived from unbalanced diets and can assist in prolonging the lifespan and provide some benefits in diseases, although evidence of direct effects is still insufficient [7,8,9].

In the last decade, the prevalence of dietary supplement use has increased dramatically [6,7], and the global market size of dietary supplements was valued at nearly 121 billion USD in 2018 [6]. The use of dietary supplements in modern society is not limited to the middle-aged and elderly, and the interest in dietary supplements is growing among young people as well. This is because most dietary supplements are not classified as drugs by the Food and Drug Administration and are easily obtainable with unrestricted exposure to advertisements. Dietary supplement intake data from the Korean National Health and Nutrition Examination Survey (KNHANES) also showed that approximately 42% of Korean adults aged ≥19 years have taken a dietary supplement at least once in their lifetime [10]. However, the role of dietary supplements is not well understood. Although the beneficial effects of dietary supplements have been shown in some studies, systematic studies on their positive and negative effects have not been reported.

In particular, if there is a problem with the function of the liver or kidney, which is the path through which dietary metabolites are absorbed and excreted, it may affect the dysfunction of these organs, and studies on the effect and safety of dietary supplements are needed. The possibility of drug–supplement interactions should also be considered.

Guidelines for CKD management recommend that patients with CKD should avoid using nutritional protein supplements and herbal remedies and should use them only under the supervision of a physician or pharmacist [11]. 

Extensive animal and clinical research have been conducted on the role of vitamin/mineral supplements and probiotics/prebiotics in kidney disease [12,13,14]. For example, vitamin D may contribute to improved clinical outcomes of CKD patients [12], and probiotics can lower the levels of inflammatory mediators and have clinical benefits in patients at different stages of CKD [13,14]. However, the association between CKD and numerous dietary supplements (e.g., ginseng and red ginseng, methyl sulfonyl methane, lutein containing supplements, propolis, and milk thistle) has not been well studied. Thus, the present study aimed to characterize the extent of dietary supplement use in a nationally representative sample and to explore the possible consequences of dietary supplements in patients with CKD.

## 2. Materials and Methods

### 2.1. Data Source and Study Population 

**Figure 1:**
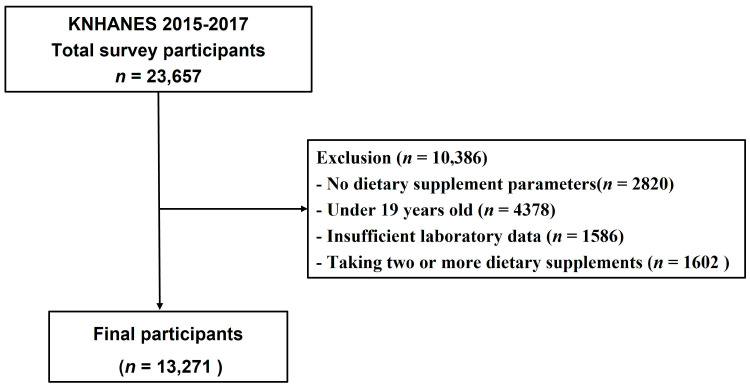
Flowchart of study population.

This study used data from the KNHANES conducted from 2015 to 2017, which is a nationwide cross-sectional sample survey conducted by the Korea Centers for Disease Control and Prevention. KNHANES includes an annual survey sample of approximately 10,000 people and consists of biochemical and clinical profiles for diseases, socioeconomic status, anthropometric measures, health-related behaviors, quality of life, health screenings, and nutritional surveys, of which the nutrition survey collects data on dietary supplements through a 24-h recall method [15]. Of the 23,657 survey participants from 2015 to 2017, 20,837 who participated in the dietary supplements survey were selected for this study (Figure 1). In addition, we excluded subjects that aged <19 years (*n* = 4378), those with missing laboratory data (*n* = 1586), and those consuming two or more dietary supplements simultaneously (*n* = 1602). The remaining 13,271 subjects were selected for this study, including 2752 subjects who used dietary supplements and 733 subjects with CKD. We defined CKD as dipstick-positive proteinuria or estimated glomerular filtration rate (eGFR) ≤ 60 mL/min/1.73 m^2^, calculated using the Chronic Kidney Disease Epidemiology Collaboration equation. 

### 2.2. Different Types of Dietary Supplements 

**Figure 2:**
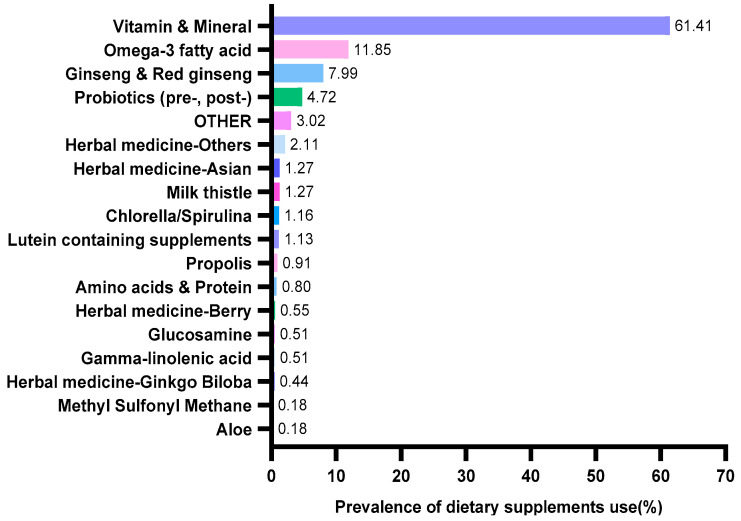
Prevalence of dietary supplements usage categories by Korean adults.

Dietary supplement data were assessed by using the following questions: “Did you consume dietary supplements over the past 24 h?” and “What is the brand name and manufacturer name of the dietary supplement that you used over the past 24 h?” According to the Health Fictional Food Code issued by the Ministry of Food and Drug Safety Notification (No. 2020-92) [16], dietary supplements were classified as aloe, amino acids and protein, gamma-linolenic acid, ginseng and red ginseng, glucosamine, herbal medicine and plant extract, lutein containing supplements, methyl sulfonyl methane, milk thistle, chlorella/spirulina, omega-3 fatty acid, probiotics (pre-, post-), propolis, and vitamin and mineral (Figure 2). Owing to the many different types of herbal medicines and plant extract used, we divided herbs into herbal medicine (plant extract) Asian, herbal medicine (plant extract) berry, herbal medicine (plant extract) ginkgo biloba, and herbal medicine (plant extract) others. Although we found 46 different dietary supplements, only the top 17 dietary supplements have been used in this study, and the rest of the dietary supplements were classified into “OTHER category (e.g., honey extract, linolenic acid dietary fiber, hyaluronic acid, placenta and collagen, squalene and alkoxyglycerol, and ursodeoxycholic acid).

### 2.3. Assessment of Covariates 

The covariates for this study included sociodemographic variables (age, sex, and education level), body mass index (BMI) (calculated as weight/height^2^), smoking status, drinking status, physical activity, systolic blood pressure (SBP), diastolic blood pressure (DBP), and medical history (hypertension and diabetes). Serum creatinine (Cr), fasting blood glucose (FBG), and triglyceride (TG) levels were measured.

### 2.4. Statistical Analyses

All statistical analyses were performed using SAS version 9.4 (SAS Institute Inc., Cary, NC, USA). Differences between continuous variables were analyzed using the independent *t*-test or Mann–Whitney U test, while categorical variables were examined using the chi-square test. Multivariable logistic regression analysis was used to analyze the association between dietary supplement consumption and CKD. Model 1 is a crude model without adjustment. Model 2 was adjusted for age and gender. Model 3 was adjusted for all variables in Model 2, as well as smoking status (smoker, ex-smoker, and non-smoker), drinking status (never, less than once a month, 1–4 times a month, and ≥5 times a month), education level (less than elementary school graduation, middle School graduation, high School graduation and college graduate or higher), physical activity (≥2 days/week, <2 days/week), SBP, DBP, BMI (<25 kg/m^2^, ≥25 kg/m^2^), FBG level, and TG level.

## 3. Results

### 3.1. General Characteristics of the Study Subjects 

Table 1 shows the characteristics of 13,271 study subjects, including 2752 in the dietary supplement group. The participants in the dietary supplement group were more likely to be women (*p* < 0.001), older adults (*p* < 0.001), non-smoker (*p* < 0.001), and highly educated individuals (*p* < 0.001). In addition, the DBP (*p* = 0.048) and serum Cr (*p* = 0.001) levels were high in the non-dietary supplement group. Among those who taking dietary supplements (Figure 2), most participants consumed vitamin and mineral (61.41%, *n* = 1690), followed by omega-3 fatty acid (11.85%, *n* = 326) and ginseng and red ginseng (7.99%, *n* = 210).

### 3.2. Association between Different Dietary Supplements and CKD

Table 2 displays dietary supplements statuses of the participants. Overall, amino acids and protein (*p* < 0.031), ginseng and red ginseng (*p* < 0.009), and herbal medicine (plant extract)-berry (*p* < 0.046) users had a higher prevalence of CKD compared to non-users. However, the CKD prevalence was lower in the group consuming probiotics than in non-user group (*p* < 0.017). Age is an important confounder; therefore, we redefined the CKD group according to the age-adapted eGFR threshold proposed in a recent study [17], and found that the prevalence of CKD was also high in the group that consumed ginseng and red ginseng (*p* < 0.03) (Supplementary Table S1). However, the above results cannot rule out the possibility that CKD patients could be overusing dietary supplements. Therefore, to clarify the direct association of dietary supplements with the development of CKD, we further analyzed dietary supplement intake in healthy individuals without a history or risk factors for CKD such as diabetes, hypertension, hyperlipidemia, and cardiovascular diseases. The results showed that the group consuming ginseng and red ginseng had a higher prevalence of CKD compared with the non-users (*p* < 0.035) (Supplementary Table S2).

In subgroup analysis according to sex, we observed that female participants who consumed amino acids and proteins (*p* < 0.009), ginseng and red ginseng (*p* < 0.007), and herbal medicine (plant extracts)-berries (*p* < 0.009) had a higher incidence of CKD than those who did not (Supplementary Table S3).

Logistic regression analysis was used to explore the association between the different dietary supplements and CKD (Table 3). After adjusting for age and sex, we observed that the prevalence of CKD was significantly high only in the herbal medicine (plant extract)-berry group (odds ratio (OR): 4.598, 95% confidence interval (CI): 1.123–18.821). Based on Model 2, we added smoking status, drinking status, education level, activity level, BMI, SBP, DBP, fasting glucose, and triglyceride for adjustment (Model 3). This result was similar to that of Model 2 (OR, 4.809; 95% CI, 1.077–21.473).

## 4. Discussion

The present large cross-sectional study assessed the extent of dietary supplement use in Korea and found that participants who consumed some dietary supplements (e.g., acids and protein, ginseng and red ginseng, and herbal medicine-berry) had a higher CKD prevalence than those who did not. In addition, the group consuming probiotics had a lower prevalence of CKD than the group that did not consume probiotics. To our knowledge, this is the first study to examine the association between dietary supplements and CKD in an Asian cohort of over 10,000 participants.

Similar to those previously reported in other studies [5,10,18,19], the most commonly consumed dietary supplements in Korea were vitamin and mineral (61.41%), omega-3 fatty acid (11.85%) and probiotics (pre-, post-) (4.72%). However, the important difference is that Koreans prefer to consume ginseng and red ginseng compared with people in other countries (7.99%). Ginseng is mainly grown in East Asian countries, and Korea is one of the world’s leading producers of ginseng [20,21]. It has been valued for its remarkable therapeutic properties. Increasing evidence has demonstrated that ginsenoside, the main component of ginseng, has antioxidant, anti-apoptotic, and inhibitory effects on inflammatory cytokines. It can also modulate blood pressure and metabolism [21,22]. The association between ginseng and red ginseng consumption and kidney disease is controversial and not fully understood. Karunasagara et al. [23] demonstrated that red ginseng showed renoprotective effects in streptozotocin-induced diabetic rats and suppressed renal inflammation and fibrosis by blocking TGF-β1 activation. Sun et al. [24] revealed that ginsenoside Rb1 can reduce renal apoptosis and alleviate renal dysfunction by activating the Nrf2/ARE signaling pathway and enhancing heme oxygenase expression. However, clinical studies have reported conflicting results. A randomized controlled trial found that long-term ginseng intake did not affect renal function in patients [25]. Additionally, other studies have reported the negative effects of ginseng consumption on the kidneys [26,27,28]. In the present study, we found that participants who consumed ginseng and red ginseng had a high prevalence of CKD. The results were the same even in a healthy population with no history or risk factors for CKD, suggesting that ginseng consumption may have a direct effect on kidney function. Although the mechanism is unknown, it is possible that ginseng has been consumed in excessive quantities, or that interactions between ginseng and drugs have had an effect. A recent study reported that ginseng has a strong interaction effect, especially in patients taking anticoagulants [29]. Considering that dietary supplementation users’ insufficient recognition of the herb-drug interactions, standards and guidelines for the safety of dietary supplements based on information about the medications taken are needed.

Probiotics, prebiotics, and synbiotics are important dietary supplements whose market has rapidly increased in recent years. Several studies have revealed that a bidirectional relationship exists between the gut microbiota and the kidney, and CKD itself can trigger dysbiosis and an altered intestinal environment. This substantial derangement is mainly caused by the decreased consumption of dietary fibers, frequent use of antibiotics, intestinal wall edema, metabolic acidosis, and uremia [30,31,32]. Recent studies have shown that dysbiosis and leaky gut in CKD are associated with an altered mucosal immune response through activation of intestinal immune cells with inflammatory cytokine production, potentially resulting in systemic inflammation and exacerbated cardiovascular/renal complications [33,34]. Therefore, improving the gut environment is considered one of the interventions to slow down the progression of CKD and prevent CKD-related complications, and probiotic consumption has attracted much attention as an adjuvant therapy to modulate gut dysbiosis. Several animal studies have demonstrated that probiotic supplements improve renal inflammation and fibrosis progression in animals with CKD [35,36]. In addition, some clinical studies have demonstrated that synbiotics have beneficial effects on improving intestinal dysbiosis and reducing serum p-cresyl sulfate levels in pre-dialysis CKD patients, and probiotic supplementation improves glucose homeostasis and systemic inflammation in dialysis patients [37,38,39]. However, owing to the limited number of studies and small sample sizes, they do not provide strong evidence for the efficacy of prebiotics or probiotics in treating CKD patients [39].

In our study, the group that consumed probiotics (pre-and post-) had a lower prevalence of CKD than the group that did not consume probiotics (pre-and post-). Given the abundance of evidence indicating the importance of kidney-gut interactions in patients with kidney disease, clinical studies are needed to demonstrate the effectiveness of microbiome-modifying therapies in large-scale CKD patients.

Modifying protein intake is an important dietary strategy for slowing CKD. Several RCTs have evaluated the effect of dietary protein restriction on renal outcomes, and overall, they suggested the benefit of dietary protein restriction. The 2020 Kidney Disease Outcomes Quality Initiative guidelines recommend dietary protein restriction in patients with metabolically stable pre-dialysis CKD to reduce disease progression or mortality [40].

According to a prospective cohort study based on the Korean Genome and Epidemiology Study conducted between 2001 and 2014, high total protein intake-induced renal hyperfiltration causes fast eGFR decline in healthy adults with normal renal function [41]. A large Italian general-population study also evaluated the effects of protein intake on serum creatinine and eGFR through questionnaire [42]. Results showed an association between protein intake and decreased renal function, and also confirmed the association between higher protein diets and eGFR levels, even excluding participants with known diabetes, hypertension or CKD. Similarly, we observed a higher prevalence of CKD among participants consuming amino acid or protein supplements. According to a recent study, the average dietary protein intake of Koreans is almost twice the estimated average requirement [43]. Therefore, additional protein supplementation can lead to an excessive protein load on the body and renal damage in several ways through increased glomerular pressure and an additional acid load on the kidney [42,44,45]. Therefore, protein supplementation should be prescribed with particular caution in patients with kidney disease, and further evaluation of the effects of prolonged exposure to high protein intake on renal function in healthy subjects is warranted.

Interestingly, we observed that consumption of herbal supplements, including berries, was associated with CKD incidence, even after adjusting for the major risk factors for the development of CKD, such as age, BMI, SBP, DBP, and fasting glucose level; this relationship was prevalent among participants with a history of CKD and CKD risk factors. Berries have conquered the global market as dietary supplements rich in vitamins, dietary prebiotic fibers, and micronutrients (e.g., zinc and iron) [46]. Morsy. et al. [47] showed that prophylactic administration of açaí berry extract in an ischemia-reperfusion animal model can improve renal function parameters and suppress the expression of renal proinflammatory cytokines and endothelin-1. Nair et al. [48] found that blueberries can protect rats with metabolic syndrome from chronic kidney injury by inhibiting Toll-like receptor 4 and attenuating mitogen-activated protein kinase activity. This renoprotective effect is attributed to the high content of flavonoids, polyphenols, and other bioactive compounds with powerful antioxidant and anti-inflammatory properties [46,49]. However, high concentrations of flavonoids in berries, especially anthocyanins, have been reported to have a cyclooxygenase (COX) inhibitory action similar to non-steroidal anti-inflammatory drugs (NSAIDs) [26,50]. NSAID-induced COX inhibition is known to be associated with CKD progression; therefore, we do not exclude the possibility that chronic use of berries may produce a similar clinical phenotype [51,52]. Another noteworthy relationship between berry consumption and kidney disease is that some berries contain a significant amount of potassium, such as 100 g of blackcurrants contain 322 mg of potassium [53], and hyperkalemia may be associated with the risk of arrhythmia or worsening of heart failure and CKD [54,55]. However, in this study, further analysis such as potassium levels or possible toxicants in berry consumers could not be performed and berries were not analyzed by type. Considering that some types of berries have a positive effect on kidney health, additional research is needed to analyze changes in kidney function after consumption of different berry types.

This study has several important advantages. Despite the steady growth of the global supplement market over the past decade, there is a dearth of information regarding the association between dietary supplements and kidney disease. This study did not simply examine the association between dietary supplements and CKD but explored as many as 17 different dietary supplements. In addition, we analyzed the general Korean population of more than 10,000 individuals using KNHANES data that are representative of the entire population. Third, to reduce the effects of the interaction between different dietary supplements, we excluded participants who consumed two or more dietary supplements from the study design. However, this study also has some limitations. First, because this was a cross-sectional study, the results cannot prove a causal relationship between CKD and dietary supplements. Secondly, since these data are only for Korean adults, the results may not be extended to other ethnic groups, and future studies in other countries and races should be conducted.

## 5. Conclusions

Our study was a large cross-sectional study that examined the association between dietary supplementation and CKD in >10,000 individuals. The study found that high incidence of CKD in the groups consuming dietary supplements, indicating that not all dietary supplements were safe for kidneys. Due to the growth of the dietary supplement market, we require special attention to the potential risk of dietary supplements to the kidney health. Furthermore, future large cohort studies are needed to provide knowledge about the precise role of each dietary supplement in CKD progression and to establish safety recommendations and guidelines for dietary supplements.

## Figures and Tables

**Table 1:** General characteristics of participants (*n* = 13,271).

	Non-Dietary Supplement Users (*n* = 10,519)		Dietary Supplements Users (*n* = 2752)		*p*
	*n* (or MEAN)	% (or STD)	*n* (or MEAN)	% (or STD)	
**Sex**					<0.0001
Male	4744	(45.10)	1051	(38.19)	
Female	5775	(54.90)	1701	(61.81)	
**Age**	50.63	±17.26	53.73	±15.68	<0.0001 ^†^
**BMI**	23.97	±3.61	23.85	±3.38	0.112
**Education**					<0.0001
Less than elementary school graduation	2280	(23.00)	580	(22.14)	
Middle School graduation	998	(10.07)	277	(10.57)	
High School graduation	3221	(32.50)	750	(28.63)	
College graduate or higher	3412	(34.43)	1013	(38.66)	
**Smoking status**					<0.0001
Smoker	1943	(18.81)	351	(12.94)	
Ex-smoker	2241	(21.69)	573	(21.12)	
Non-smoker	6148	(59.50)	1789	(65.94)	
**Drinking status**					<0.0001
Never	1220	(11.80)	341	(12.56)	
Less than once a month	3438	(33.25)	1002	(36.89)	
1–4 times a month	3341	(32.31)	867	(31.92)	
≥5 times a month	2341	(22.64)	506	(18.63)	
**Physical activity**					0.311
<2 days/week	9097	(91.70)	2391	(91.09)	
≥2 days/week	823	(8.30)	234	(8.91)	
**Diabetes**					0.843
No	9319	(90.33)	2439	(90.20)	
Yes	998	(9.67)	265	(9.80)	
**Hypertension**					<0.0001
No	7845	(76.02)	1957	(72.35)	
Yes	2474	(23.98)	748	(27.65)	
**CKD**					0.851
No	9940	(94.50)	2598	(94.40)	
Yes	579	(5.50)	154	(5.60)	
**SBP**	119.06	±16.95	118.95	±16.74	0.762
**DBP**	75.19	±10.20	74.76	±9.96	0.048
**Cr**	0.84	±0.36	0.82	±0.26	0.001 ^†^
**FBG**	101.69	±26.69	101.15	±24.15	0.541 ^†^
**TG**	136.70	±116.63	133.19	±95.17	0.216 ^†^

Values are presented as N (%) or mean ± STD. *p*-values were calculated using the independent *t*-test for normally distributed continuous variables, Mann–Whitney U test for non-normally distributed continuous variables, and Chi-square test for categorical variables. ^†^ Mann–Whitney U test. CKD: chronic kidney disease; N: number; STD: standard deviation; BMI: body mass index; SBP: systolic blood pressure; DBP: diastolic blood pressure; Cr: creatinine; FBG: fasting blood glucose; TG: triglyceride.

**Table 2:** Dietary supplements statuses of the participants (***n*** = 13,271).

	CKD (*n* = 733)		No CKD (*n* = 12,538)		
	n	%	n	%	*p*
**Aloe**					>0.999
Yes	0	(0.00)	5	(100.00)	
No	733	(5.53)	12533	(94.47)	
**Amino acids and Protein**					0.031
Yes	4	(18.18)	18	(81.82)	
No	729	(5.50)	12,520	(94.50)	
**Chlorella/Spirulina**					0.696
Yes	2	(6.25)	30	(93.75)	
No	731	(5.52)	12,508	(94.48)	
**Gamma-linolenic acid**					>0.999
Yes	0	(0.00)	14	(100.00)	
No	733	(5.53)	12,524	(94.47)	
**Ginseng and Red ginseng**					0.009
Yes	21	(9.55)	199	(90.45)	
No	712	(5.46)	12,339	(94.54)	
**Glucosamine**					0.549
Yes	1	(7.14)	13	(92.86)	
No	732	(5.52)	12,525	(94.48)	
**Herbal medicine (plant extract) Asian**					0.720
Yes	2	(5.71)	33	(94.29)	
No	731	(5.52)	12,505	(94.48)	
**Herbal medicine (plant extract) Berry**					0.046
Yes	3	(20.00)	12	(80.00)	
No	730	(5.51)	12,526	(94.49)	
**Herbal medicine (plant extract) Ginkgo Biloba**					>0.999
Yes	0	(0.00)	12	(100.00)	
No	733	(5.53)	12,526	(94.47)	
**Herbal medicine (plant extract) Others**					0.378
Yes	1	(1.72)	57	(98.28)	
No	732	(5.54)	12,481	(94.46)	
**Lutein containing supplements**					0.689
Yes	2	(6.45)	29	(93.55)	
No	731	(5.52)	12,509	(94.48)	
**Methyl Sulfonyl Methane**					>0.999
Yes	0	(0.00)	5	(100.00)	
No	733	(5.53)	12,533	(94.47)	
**Milk thistle**					0.262
Yes	0	(0.00)	35	(100.00)	
No	733	(5.54)	12,503	(94.46)	
**Omega-3 fatty acid**					0.050
Yes	26	(7.98)	300	(92.02)	
No	707	(5.46)	12,238	(94.54)	
**Probiotics (pre-, post-)**					0.017
Yes	1	(0.77)	129	(99.23)	
No	732	(5.57)	12,409	(94.43)	
**Propolis**					>0.999
Yes	1	(4.00)	24	(96.00)	
No	732	(5.53)	12,514	(94.47)	
**Vitamin and Mineral**					0.470
Yes	87	(5.15)	1603	(94.85)	
No	646	(5.58)	10,935	(94.42)	

Values are presented as N (%) for categorical variables, and *p*-values were calculated using the chi-square test or Student’s *t*-test. CKD: chronic kidney disease.

**Table 3:** Odds ratio (OR) of the incidence of CKD according to take different dietary supplements.

	Model 1			Model 2			Model 3		
	OR	95% CI	*p*	OR	95% CI	*p*	OR	95% CI	*p*
Amino acids and Protein	3.817	(1.288–11.306)	0.016	2.199	(0.699–6.915)	0.178	2.291	(0.723–7.257)	0.159
Ginseng and Red ginseng	1.829	(1.159–2.886)	0.010	1.338	(0.825–2.169)	0.238	1.35	(0.817–2.232)	0.241
Herbal medicine (plant extract) Berry	4.290	(1.208–15.235)	0.024	4.598	(1.123–18.821)	0.034	4.809	(1.077–21.473)	0.040
Probiotics (pre-, post-)	0.132	(0.018–0.941)	0.043	0.207	(0.028–1.510)	0.120	0.237	(0.032–1.741)	0.157
Omega-3 fatty acid	1.500	(0.998–2.256)	0.051	1.015	(0.666–1.547)	0.945	1.019	(0.655–1.584)	0.933
Chlorella/Spirulina	1.141	(0.272–4.783)	0.857	1.186	(0.262–5.364)	0.825	1.151	(0.244–5.420)	0.859
Glucosamine	1.317	(0.172–10.076)	0.791	0.528	(0.067–4.187)	0.546	0.591	(0.074–4.723)	0.620
Herbal medicine (plant extract) Asian	1.037	(0.249–4.330)	0.960	0.820	(0.188–3.585)	0.792	1.121	(0.254–4.940)	0.880
Herbal medicine (plant extract) Others	0.300	(0.042–2.164)	0.233	0.628	(0.085–4.630)	0.648	0.759	(0.102–5.625)	0.787
Lutein containing supplements	1.180	(0.281–4.956)	0.821	1.287	(0.287–5.769)	0.741	1.424	(0.292–6.939)	0.662
Propolis	0.713	(0.096–5.273)	0.740	0.879	(0.110–7.008)	0.903	1.233	(0.151–10.073)	0.845
Vitamin and Mineral	0.919	(0.730–1.156)	0.470	0.907	(0.714–1.153)	0.426	0.980	(0.762–1.260)	0.874

Model 1: unadjusted; Model 2: adjusted for age and sex; Model 3: Model 2 + smoking status, drinking status, education level, BMI, SBP, DBP, fasting blood glucose, and triglyceride; OR: odds ratio; CI: confidence interval.

## Data Availability

The Korea National Health and Nutrition Examination Survey (KNHANES) data are publicly available on the official KNHANES website (https://knhanes.kdca.go.kr, accessed on 19 November 2022).

## Supplementary Materials

The following supporting information can be downloaded at: https://www.mdpi.com/article/10.3390/nu15040822/s1, Supplementary Table S1. General participant characteristics Supplementary Table S2. General characteristics of participants with and without a history of CKD or risk factors for CKD. Supplementary Table S3. General characteristics of participants according to sex.
