# Supplementary material for: Association between Consumption of Dietary Supplements and Chronic Kidney Disease Prevalence: Results of the Korean Nationwide Population-Based Survey
Source: Nutrients. 2023 Feb 5;15(4):822. doi: 10.3390/nu15040822 (PMC9967330; doi:10.3390/nu15040822)
Supplement: Supplementary file 1 [file nutrients-15-00822-s001.zip › nutrients-2188711-supplementary.pdf]

Supplementary Table S1. General participant characteristics

Types of dietary supplement	CKD (n = 396)		Normal (n = 12,875)		
	n	%	n	%	p-value
Aloe					>.999
Yes	0	(0.00)	5	(100.00)	
No	396	(2.99)	12870	(97.01)	
Amino acids & Protein					0.487
Yes	1	(4.55)	21	(95.45)	
No	395	(2.98)	12854	(97.02)	
Chlorella/Spirulina					0.621
Yes	1	(3.13)	31	(96.88)	
No	395	(2.98)	12844	(97.02)	
Gamma-linolenic acid					>.999
Yes	0	(0.00)	14	(100.00)	
No	396	(2.99)	12861	(97.01)	
Ginseng & Red ginseng					0.030
Yes	12	(5.45)	208	(94.55)	
No	384	(2.94)	12667	(97.06)	
Glucosamine					>.999
Yes	0	(0.00)	14	(100.00)	
No	396	(2.99)	12861	(97.01)	
Herbal medicine (plant extract) Asian					0.281
Yes	2	(5.71)	33	(94.29)	
No	394	(2.98)	12842	(97.02)	
Herbal medicine (plant extract) Berry					0.072
Yes	2	(13.33)	13	(86.67)	
No	394	(2.97)	12862	(97.03)	
Herbal medicine (plant extract) Ginkgo Biloba					>.999
Yes	0	(0.00)	12	(100.00)	
No	396	(2.99)	12863	(97.01)	
Herbal medicine (plant extract) Others					>.999
Yes	1	(1.72)	57	(98.28)	
No	395	(2.99)	12818	(97.01)	
Lutein containing supplements					0.420
Yes	5	(4.07)	118	(95.93)	
No	391	(2.97)	12757	(97.03)	
Methyl Sulfonyl Methane					>.999
Yes	0	(0.00)	31	(100.00)	
No	396	(2.99)	12844	(97.01)	
Milk thistle					>.999
Yes	0	(0.00)	5	(100.00)	
No	396	(2.99)	12870	(97.01)	
Omega-3 fatty acid					0.627
Yes	0	(0.00)	35	(100.00)	
No	396	(2.99)	12840	(97.01)	
Probiotics (pre-, post-)					0.281
Yes	13	(3.99)	313	(96.01)	
No	383	(2.96)	12562	(97.04)	
Propolis					0.191
Yes	1	(0.77)	129	(99.23)	
No	395	(3.01)	12746	(96.99)	
Vitamin & Mineral					>.999
Yes	0	(0.00)	25	(100.00)	
No	396	(2.99)	12850	(97.01)	

The data are shown as N (%) for categorical variables, and p-values were calculated using the chi-square test or Student's t-test. CKD, chronic kidney disease (CKD was defined by eGFR threshold of 75, 60, and 45 mL/min/1.73 m^2 for younger than 40, 40 to 64, and 65 years or older, respectively, or a positive dipstick urinalysis).

Supplementary Table S2. General characteristics of participants with and without a history of CKD or risk factors for CKD.

Types of dietary supplement	Healthy population (*n* = 8,164)					Unhealthy population (*n* = 4,568)				
	CKD (*n* = 125)		Normal (*n* = 8,039)			CKD (*n* = 582)		Normal (*n* = 3,986)		
	n	%	n	%	p	n	%	n	%	p
Aloe					>.999					>.999
Yes	0	(0.00)	4	(100.00)		0	(0.00)	1	(100.00)	
No	125	(1.53)	8035	(98.47)		582	(12.74)	3985	(87.26)	
Amino acids & Protein					>.999					0.073
Yes	0	(0.00)	8	(100.00)		4	(30.77)	9	(69.23)	
No	125	(1.53)	8031	(98.47)		578	(12.69)	3977	(87.31)	
Chlorella/Spirulina					>.999					0.321
Yes	0	(0.00)	22	(100.00)		2	(22.22)	7	(77.78)	
No	125	(1.54)	8017	(98.46)		580	(12.72)	3979	(87.28)	
Gamma-linolenic acid					>.999					>.999
Yes	0	(0.00)	7	(100.00)		0	(0.00)	4	(100.00)	
No	125	(1.53)	8032	(98.47)		582	(12.75)	3982	(87.25)	
Ginseng & Red ginseng					0.035					0.322
Yes	5	(4.24)	113	(95.76)		15	(16.13)	78	(83.87)	
No	120	(1.49)	7926	(98.51)		567	(12.67)	3908	(87.33)	
Glucosamine					>.999					>.999
Yes	0	(0.00)	6	(100.00)		1	(14.29)	6	(85.71)	
No	125	(1.53)	8033	(98.47)		581	(12.74)	3980	(87.26)	
Herbal medicine (plant extract) Asian					>.999					>.999
Yes	0	(0.00)	20	(100.00)		2	(13.33)	13	(86.67)	
No	125	(1.53)	8019	(98.47)		580	(12.74)	3973	(87.26)	
Herbal medicine (plant extract) Berry					>.999					0.031
Yes	0	(0.00)	9	(100.00)		3	(50.00)	3	(50.00)	
No	125	(1.53)	8030	(98.47)		579	(12.69)	3983	(87.31)	
Herbal medicine (plant extract) Ginkgo Biloba					>.999					>.999
Yes	0	(0.00)	6	(100.00)		0	(0.00)	6	(100.00)	
No	125	(1.53)	8033	(98.47)		582	(12.76)	3980	(87.24)	
Herbal medicine (plant extract) Others					>.999					>.999
Yes	0	(0.00)	46	(100.00)		1	(9.09)	10	(90.91)	
No	125	(1.54)	7993	(98.46)		581	(12.75)	3976	(87.25)	
Lutein containing supplements					>.999					0.627
Yes	0	(0.00)	17	(100.00)		6	(15.38)	33	(84.62)	
No	125	(1.53)	8022	(98.47)		576	(12.72)	3953	(87.28)	
Methyl Sulfonyl Methane					>.999					0.658
Yes	0	(0.00)	4	(100.00)		2	(16.67)	10	(83.33)	
No	125	(1.53)	8035	(98.47)		580	(12.73)	3976	(87.27)	
Milk thistle					>.999					>.999
Yes	0	(0.00)	23	(100.00)		0	(0.00)	1	(100.00)	
No	125	(1.54)	8016	(98.46)		582	(12.74)	3985	(87.26)	
Omega-3 fatty acid					0.041					0.379
Yes	5	(4.03)	119	(95.97)		0	(0.00)	11	(100.00)	
No	120	(1.49)	7920	(98.51)		582	(12.77)	3975	(87.23)	

Probiotics (pre-, post-)					0.407					0.337
Yes	0	(0.00)	92	(100.00)		20	(10.47)	171	(89.53)	
No	125	(1.55)	7947	(98.45)		562	(12.84)	3815	(87.16)	
Propolis					0.254					0.117
Yes	1	(5.26)	18	(94.74)		1	(2.94)	33	(97.06)	
No	124	(1.52)	8021	(98.48)		581	(12.81)	3953	(87.19)	
Vitamin & Mineral					0.490					>.999
Yes	13	(1.28)	1001	(98.72)		0	(0.00)	5	(100.00)	
No	112	(1.57)	7038	(98.43)		582	(12.75)	3981	(87.25)	

The data are shown as N (%) for categorical variables, and p-values were calculated using the chi-square test or Student's t-test. CKD: chronic kidney disease

Supplementary Table S3. General characteristics of the participants according to sex

	Male (*n* = 5,795)					Female (*n* = 7,476)				
**Types of dietary supplement**	**CKD (*n* = 389)**		**Normal (*n* = 5,406)**			**CKD (*n* = 344)**		**Normal (*n* = 7,132)**		
	**n**	**%**	**n**	**%**	**p**	**n**	**%**	**n**	**%**	**p**
Aloe					>.999					>.999
Yes	0	(0.00)	2	(100.00)		0	(0.00)	3	(100.00)	
No	389	(6.72)	5404	(93.28)		344	(4.60)	7129	(95.40)	
Amino acids & Protein					0.566					0.009
Yes	1	(8.33)	11	(91.67)		3	(30.00)	7	(70.00)	
No	388	(6.71)	5395	(93.29)		341	(4.57)	7125	(95.43)	
Chlorella/Spirulina					0.465					>.999
Yes	1	(11.11)	8	(88.89)		1	(4.35)	22	(95.65)	
No	388	(6.71)	5398	(93.29)		343	(4.60)	7110	(95.40)	
Gamma-linolenic acid					-					>.999
Yes	0	(0.00)	0	(0.00)		0	(0.00)	14	(100.00)	
No	389	(6.71)	5406	(93.29)		344	(4.61)	7118	(95.39)	
Ginseng & Red ginseng					0.278					0.007
Yes	9	(9.47)	86	(90.53)		12	(9.60)	113	(90.40)	
No	380	(6.67)	5320	(93.33)		332	(4.52)	7019	(95.48)	
Glucosamine					>.999					0.376
Yes	0	(0.00)	4	(100.00)		1	(10.00)	9	(90.00)	
No	389	(6.72)	5402	(93.28)		343	(4.59)	7123	(95.41)	
Herbal medicine (plant extract) Asian					0.465					>.999
Yes	1	(11.11)	8	(88.89)		1	(3.85)	25	(96.15)	
No	388	(6.71)	5398	(93.29)		343	(4.60)	7107	(95.40)	
Herbal medicine (plant extract) Berry					>.999					0.009
Yes	0	(0.00)	5	(100.00)		3	(30.00)	7	(70.00)	
No	389	(6.72)	5401	(93.28)		341	(4.57)	7125	(95.43)	
Herbal medicine (plant extract) Ginkgo Biloba					>.999					>.999
Yes	0	(0.00)	1	(100.00)		0	(0.00)	11	(100.00)	
No	389	(6.71)	5405	(93.29)		344	(4.61)	7121	(95.39)	
Herbal medicine (plant extract) Others					0.629					>.999
Yes	0	(0.00)	18	(100.00)		1	(2.50)	39	(97.50)	
No	389	(6.73)	5388	(93.27)		343	(4.61)	7093	(95.39)	
Lutein containing supplements					0.465					>.999
Yes	1	(11.11)	8	(88.89)		1	(4.55)	21	(95.45)	
No	388	(6.71)	5398	(93.29)		343	(4.60)	7111	(95.40)	
Methyl Sulfonyl Methane					>.999					>.999
Yes	0	(0.00)	1	(100.00)		0	(0.00)	4	(100.00)	
No	389	(6.71)	5405	(93.29)		344	(4.60)	7128	(95.40)	
Milk thistle					0.257					>.999
Yes	0	(0.00)	28	(100.00)		0	(0.00)	7	(100.00)	
No	389	(6.75)	5378	(93.25)		344	(4.61)	7125	(95.39)	
Omega-3 fatty acid					0.006					0.928
Yes	17	(12.50)	119	(87.50)		9	(4.74)	181	(95.26)	
No	372	(6.57)	5287	(93.43)		335	(4.60)	6951	(95.40)	

Probiotics (pre-, post-)					0.257					0.092
Yes	0	(0.00)	28	(100.00)		1	(0.98)	101	(99.02)	
No	389	(6.75)	5378	(93.25)		343	(4.65)	7031	(95.35)	
Propolis					>.999					0.530
Yes	0	(0.00)	9	(100.00)		1	(6.25)	15	(93.75)	
No	389	(6.72)	5397	(93.28)		343	(4.60)	7117	(95.40)	
Vitamin & Mineral					0.855					0.572
Yes	43	(6.54)	614	(93.46)		44	(4.26)	989	(95.74)	
No	346	(6.73)	4792	(93.27)		300	(4.66)	6143	(95.34)	

The data are shown as N (%) for categorical variables, and p-values were calculated using the chi-square test or Student's t-test. CKD: chronic kidney disease
